# New and little-known bees of the genus *Colletes* Latreille, 1802 (Hymenoptera, Colletidae) from the Nakhchivan Autonomous Republic of Azerbaijan, with descriptions of two new species

**DOI:** 10.3897/zookeys.1268.174008

**Published:** 2026-02-04

**Authors:** Michael Kuhlmann, Maxim Yu. Proshchalykin, Mahir M. Maharramov

**Affiliations:** 1 Zoological Museum of Kiel University, Hegewischstr. 3, D-24105 Kiel, Germany Nakhchivan State University Nakhchivan Azerbaijan; 2 Federal Scientific Center of the East Asia Terrestrial Biodiversity, Far East Branch of the Russian Academy of Sciences, Vladivostok 690022, Russia Zoological Museum of Kiel University Kiel Germany; 3 Nakhchivan State University, University Campus, AZ 7012 Nakhchivan, Azerbaijan Federal Scientific Center of the East Asia Terrestrial Biodiversity, Far East Branch of the Russian Academy of Sciences Vladivostok Russia

**Keywords:** Anthophila, Apiformes, Caucasus, Palaearctic region, taxonomy

## Abstract

Data on 14 species of the genus *Colletes* Latreille, 1802 collected in the Nakhchivan Autonomous Republic of Azerbaijan in 2018–2025 are provided. Among them, *Colletes perplexus***sp. nov**. is described as new to science. *Colletes caskanus* (Strand, 1919), *C. glaber* Warncke, 1978 and *C. idoneus* Cockerell, 1922 are newly recorded from Azerbaijan and ten species are newly recorded from the Nakhchivan Autonomous Republic. Now 43 species of *Colletes* are known from Azerbaijan. As a result of its first discovery in Azerbaijan, the intraspecific variation of the variable *C. glaber* was examined through its range and the new species *C. quadratus* Kuhlmann, **sp. nov**. from southeastern Turkey is described.

## Introduction

The present paper is a continuation of the ongoing research of the bees from the Nakhchivan Autonomous Republic of Azerbaijan (Aliyev et al. 2017; Proshchalykin et al. 2019; Fateryga et al. 2020, 2025; Proshchalykin and Maharramov 2020; Proshchalykin and Dathe 2021; Maharramov et al. 2021, 2023; Astafurova et al. 2024a, b). The Nakhchivan Autonomous Republic is an exclave of Azerbaijan, located in the southern part of the Transcaucasian plateau. It is bounded by Armenia to the north and east, Iran to the south and west, and Turkey to the west. The republic, which is mostly mountainous except for a plain in the west and southwest, lies to the east and north of the middle Aras River, which forms the frontier with Iran and Turkey. Currently more than 350 bee species are known from this territory, but the *Colletes* fauna of the Nakhchivan Autonomous Republic is particularly understudied.

The genus *Colletes* Latreille, 1802 currently includes approximately 520 described species with an estimated total of ~ 700 species (Proshchalykin and Kuhlmann 2023) from all continents except Antarctica and Oceania (Michener 2007; Kuhlmann 2014). So far 39 species have been recorded from Azerbaijan and only eleven species from the Nakhchivan Autonomous Republic (Kuhlmann and Proshchalykin 2015, 2016; Proshchalykin and Kuhlmann 2018).

Based on a study of recently collected specimens we here provide additional geographical data for 14 rarely collected and little-known species of *Colletes*, with one species described as new and three species recorded from Azerbaijan for the first time; new distributional records of ten *Colletes* species from the Nakhchivan Autonomous Republic are also reported. The number of *Colletes* species in the fauna of Azerbaijan now increased to 43.

As a result of its first discovery in Azerbaijan, the intraspecific variation of the variable *Colletes glaber* Warncke, 1978 was examined through its range and the new species *C. quadratus* Kuhlmann, sp. nov. from southeastern Turkey is described.

## Materials and methods

Terminology as well as measurements used in the descriptions follow those of Michener (2007). Puncture density is expressed as the relationship between puncture diameter (**d**) and the space between them (**i**), such as i = 1.5d or i < d. The letter **T** is used as abbreviation of metasomal tergum, the letter **S** for a metasomal sternum and the letter **F** for antennal flagellar segment. Body length is measured from the vertex to the apex of the metasoma. The definition of species groups in *Colletes* follows Noskiewicz (1936) and Kuhlmann et al. (2009). Species are listed in alphabetical order.

Images were taken with the Digital Microscope Keyence VHX-5000 (Keyence Deutschland GmbH, Neu-Isenburg, Germany) using the VH-Z20R/Z20T (20–200× zoom lens and the OP-42305 super diffused illumination adapter. Photoshop elements (Adobe Systems Software Ireland Limited, Dublin, Republic of Ireland) were used for image processing.

Missing coordinates on the original specimen labels were identified using Google Earth (Google Earth Pro© 2021, v. 7.3.4.8248) and added in [square brackets] (format degree.minutes). The distribution map was created with SimpleMappr (Shorthouse 2010).

Specimens were examined from the following collections: **AMNH** – American Museum of Natural History, New York, USA; **FSCV** – Federal Scientific Center of the East Asia terrestrial biodiversity, Vladivostok, Russia (registration number 2797657); **OÖLM** – Biodiversity Center Linz (Oberösterreichisches Landesmuseum Linz), Austria; **NBCL** – Naturalis Biodiversity Center, Leiden, Netherlands (formerly in Coll. Zoölogisch Museum Amsterdam); **PCES** – personal collection of Erwin Scheuchl, Ergolding, Germany; **PCMS** – personal collection of Maximilian Schwarz, Ansfelden, Austria; **RCMK** – research collection of Michael Kuhlmann, Zoological Museum of Kiel University, Germany; **ZISP**—Zoological Institute of the Russian Academy of Sciences, Saint Petersburg, Russia.

The results presented in this paper are based on 98 *Colletes* specimens collected in the Nakhchivan Autonomous Republic of Azerbaijan in 2018–2025. We have used the following abbreviations for collectors: **HA** – H. Aliyev; **MP** – M. Proshchalykin; **MM** – M. Maharramov. Distribution of species generally follows Kuhlmann and Proshchalykin (2016) and Proshchalykin (2017). New distributional records are noted with an asterisk (*).

## Taxonomy

### Genus Colletes

Taxon classificationAnimaliaHymenopteraColletidae

Latreille, 1802

4BF2FDA4-C925-5C8F-B5CD-183FBF3D17F6

Colletes Latreille, 1802: 423. Type species: Apis succincta Linnaeus, 1758, by monotypy.

### *Colletes hylaeiformis* species group

#### Colletes hylaeiformis

Taxon classificationAnimaliaHymenopteraColletidae

Eversmann, 1852

AEE1FEEE-AF64-5B9B-94A9-EDB7C59D71F3

Colletes hylaeiformis Eversmann, 1852: 4, ♀, ♂. Lectotype: ♀, Volga inf. [Astrakhan Province, Russia], designated by Proshchalykin 2017: 32.

##### Material examined.

**Azerbaijan. Nakhchivan Autonomous Republic** • Ordubad, Aghdara, 39°06'N, 45°54'E, 28.VII.2018, 3 ♂, MP, HA, MM [FSCV/RCMK].

##### Distribution.

Europe, Russia (North Caucasus, European part), Georgia, Azerbaijan (including *Nakhchivan Autonomous Republic), Kazakhstan, Uzbekistan, Tajikistan.

### *Colletes nigricans* species group

#### Colletes eous

Taxon classificationAnimaliaHymenopteraColletidae

Morice, 1904

29D87A14-2173-54C6-A60B-6AB03AEFE877

Colletes eous Morice, 1904: 43–44, ♀, ♂. Syntypes: ♀♀, ♂♂, Helenendorf, Azerbaijan.

##### Material examined.

**Azerbaijan. Nakhchivan Autonomous Republic** • Shakhbuz, Batabat, 39°31'N, 45°47'E, 24.VII.2018, 3 ♂, MP, HA, MM [FSCV].

##### Distribution.

North Africa, Europe, Russia (North Caucasus, European part), Georgia, Azerbaijan (including *Nakhchivan Autonomous Republic), Turkey, Lebanon, Iran, Central Asia, India.

### *Colletes caspicus* species group

#### Colletes idoneus

Taxon classificationAnimaliaHymenopteraColletidae

Cockerell, 1922

4D350DF8-0515-5433-819A-17BA3EBABEEC

Colletes idoneus Cockerell, 1922: 363, ♂. Holotype: ♂, Quetta, Baluchistan, Pakistan.

##### Material examined.

**Azerbaijan. Nakhchivan Autonomous Republic** • Kangarli, Chalkhangala, 39°26'N, 45°17'E, 1370 m, 17.VI.2020, 1 ♂, MM [FSCV].

##### Distribution.

Armenia, *Azerbaijan (Nakhchivan Autonomous Republic), Iran, Pakistan, Tajikistan, Turkmenistan.

### Colletes maidli

Taxon classificationAnimaliaHymenopteraColletidae

Noskiewicz, 1936

1DFB1573-A4AD-51E6-BB8A-5F49A35F825C

Colletes maidli Noskiewicz, 1936: 166–168, ♀, ♂. Syntypes: ♀♀, ♂♂, Italy, Spain, Syria, Azerbaijan.

#### Material examined.

**Azerbaijan. Nakhchivan Autonomous Republic** • Julfa, Gazanchi, 39°13'N, 45°41'E, 27.VII.2018, 1 ♀, MP, HA, MM [FSCV]; • Babek, Shikhmakhmud, 39°15'N, 45°25'E, 940 m, 24.VI.2021, 1 ♂, MM [FSCV]; • Julfa, Milakh, 39°15'N, 45°43'E, 1430 m, 24.VI.2024, 1 ♂, MP, MM [FSCV].

#### Distribution.

North Africa, Europe, Russia (North Caucasus, European part), Georgia, Azerbaijan (including *Nakhchivan Autonomous Republic), Turkey, Syria, Israel, Iran, Kazakhstan.

##### *Colletes squamosus* species group

### Colletes wollmanni

Taxon classificationAnimaliaHymenopteraColletidae

Noskiewicz, 1936

532C206C-B9AF-5770-AC7A-C15E7C4B04C5

Colletes wollmanni Noskiewicz, 1936: 188–191, ♀, ♂. Lectotype: ♂, Baigakum bei Djulek, Turkest. [Kazakhstan], designated by Proshchalykin and Kuhlmann 2015: 550.

#### Material examined.

**Azerbaijan. Nakhchivan Autonomous Republic** • Ordubad, Bilav, 39°02'N, 45°49'E, 1050 m, 29.V.2025, 1 ♂, MP, MM [FSCV].

#### Distribution.

Russia (North Caucasus), Azerbaijan (including *Nakhchivan Autonomous Republic), Iran, Pakistan, Central Asia, China.

##### *Colletes nanus* species group

### Colletes perplexus sp. nov.

Taxon classificationAnimaliaHymenopteraColletidae

68390FDE-24E5-5959-913F-4AC3F0D5A155

https://zoobank.org/E0C49976-4E94-460D-8FAF-1BA9423DB6CC

Fig. 1A–F

#### Type material (3 specimens).

***Holotype***: ♂. **Azerbaijan**. • “Azerbaijan, Nakhchivan AR, Ordubad, Kotam, 700 m, 38°53'25"N, 46°03'14"E, 19.V.2025, leg. Proshchalykin, Maharramov” (ZISP). ***Paratypes*. Azerbaijan**. • “Azerbaijan, Nakhchivan AR, Ordubad, Bilav, 1050 m, 39°02'43"N, 45°49'07"E, 22.V.2025, 2 ♂ Proshchalykin, Maharramov” (FSCV/RCMK).

**Figure 1.**
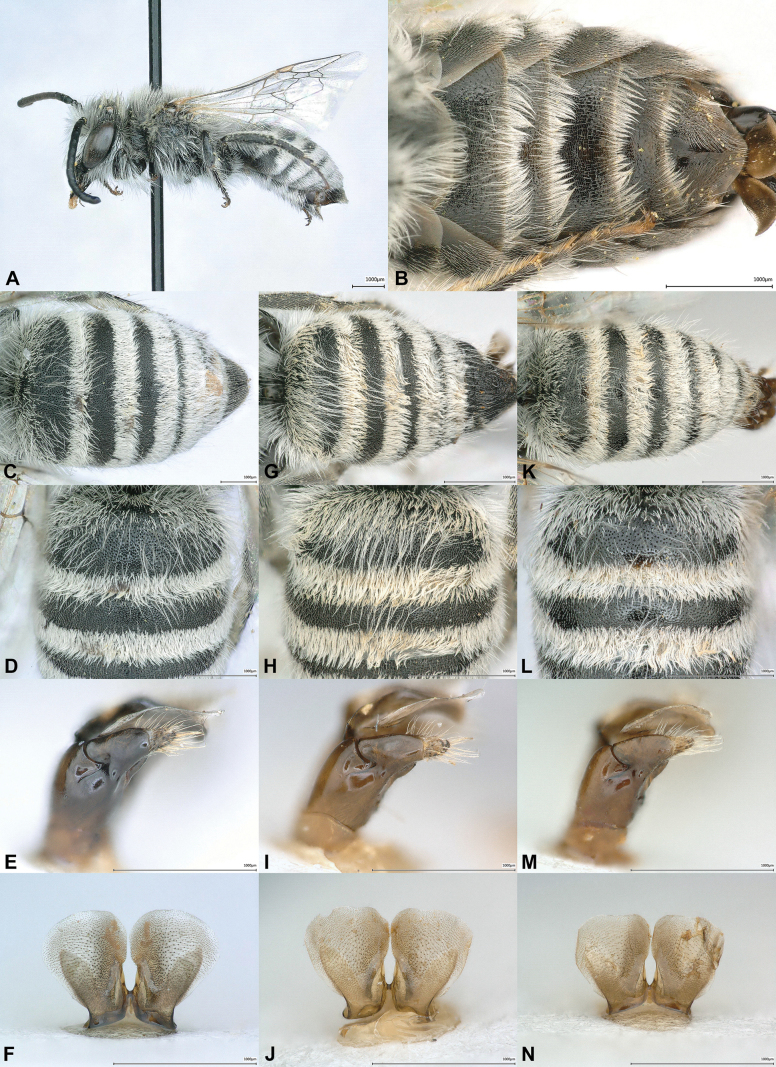
Males of *Colletes perplexus* sp. nov. (**A–F**), *C. schwarzi* Kuhlmann, 2002 (**G–J**) and *C. popovi* Noskiewicz, 1936 (**K–N**). **A**. Habitus, lateral view; **B**. Metasoma, ventral view; **C, G, K**. Metasoma, dorsal view; **D, H, L**. T1–T2, dorsal view; **E, I, M**. Gonostylus, lateral view. **F, J, N**. S7, dorsal view.

#### Diagnosis.

The new species belongs to the *C. nanus* species group that is characterised in the male sex by overall small size (maximum body length ~9.0 mm), malar area elongate, mesoscutal disc polished, either impunctate or sparsely punctate, anterior declivorous part of T1 densely covered with short appressed and long erect hair, T1–T5 and S2–S5 with dense and broad white apical hair bands (Noskiewicz 1936). This group is particularly speciose in deserts and semi-deserts of North Africa and the Middle East. Far fewer species occur in the Caucasus region and Middle Asia, namely *C. popovi* Noskiewicz, 1936, *C. schwarzi* Kuhlmann, 2002 and the enigmatic *C. penulatus* Noskiewicz, 1936. The latter species is known only from the single female holotype and, thus, is not considered here (but see comment below under Remarks). In neighbouring Iran, *C. sirjanensis* Kuhlmann, 2023 was described based on males but its oval-shaped S7 (Kuhlmann et al. 2023) differs significantly from the ± round S7 of *C. perplexus*, *C. schwarzi* and *C. popovi*, hence, it is also not included here. Therefore, the diagnosis is limited to these three closely related species, which are the only others of the *C. nanus* species group found in the region and share a very similar morphology. They are extensively illustrated in this paper to facilitate their identification.

The recently described *C. storozhenkoi* Kuhlmann & Proshchalykin, 2025 (Proshchalykin and Kuhlmann 2025) is also not considered here. The species is also known only from females collected at a single site in Dagestan in the coastal lowlands of the Caspian Sea and the following morphological features suggests that *C. storozhenkoi* and *C. perplexus* very likely represent two separate taxa. In *C. storozhenkoi* punctation on the mesoscutal disc unusually dense for a species in this group (much more dispersed in *C. perplexus*), hair on mesoscutum in particular posteriorly short and thickly plumose (*C. perplexus*: disproportionately longer, more slender, and less plumose), horizontal part of propodeum shorter (*C. perplexus*: disproportionately longer) and depressions of apical tergal margins differ disproportionally for sexes of the same species. However, this can only be clarified ultimately if the unknown sex of at least one of the two species is found or through genetic analysis (e.g. DNA barcoding), which was not feasible within the context of this study.

The male of *C. perplexus* differs from *C. popovi* and *C. schwarzi* by the following character combination: Apical tergal hair bands narrower (Fig. 1C) than in the other species (Fig. 1G, K), appressed hair on the sloping base of T1 sparser and more restricted, barely reaching the disc (Fig. 1D) (appressed hair denser and more extensive in *C. schwarzi* and *C. popovi* as in Fig. 1H, L), punctation on discs of T1 and T2 fine and dense (i < d) (Fig. 1D) (in *C. popovi* more dispersed (i = 1–2d) as in Fig. 1L), apical sternal hair fringes medially significantly shorter than laterally (Fig. 1B) (in *C. popovi* fringes only slightly shorter medially), S7 overall and especially apically more evenly rounded (Fig. 1F) while apically rather flattened or a bit emarginate in *C. schwarzi* and *C. popovi* (Fig. 1J, N), gonostylus basally slightly broader and of different shape (Fig. 1E) than in the other two species (Fig. 1I, M).

#### Description.

**Female**. Unknown.

**Male**. Body length = 7.0–8.0 mm. ***Head***. Head wider than long (width to length ratio: 1.3: 1). Integument black except tip of mandible partly dark reddish-brown. Face densely covered with long, white to yellowish-brown, erect hairs; on vertex partly darker brown. Malar area medially ~ 3/4 as long as width of mandible base, finely striate. Antenna dorsally dark blackish-brown to black, ventrally slightly lighter; F1 as long as wide (length to width ratio: 1:1). ***Mesosoma***. Integument black. Dorsolateral angle of pronotum rectangular and rounded. Mesoscutal disc sparsely punctate (i = 1–3d), interspace smooth and shiny. Scutellum anteriorly almost impunctate, smooth and shiny, posteriorly densely punctate. Mesoscutum, scutellum, metanotum, mesepisternum and propodeum covered with long, yellowish-brown, laterally white erect hair (Fig. 1A). ***Wings***. Transparent, membrane very slightly light yellow; wing venation yellowish-brown to blackish. ***Legs***. Integument black; last tarsal segment at least partly, usually completely brown. Hind basitarsus ~ 4× as long as wide. Vestiture white to yellowish-white (Fig. 1A). ***Metasoma***. Integument black; depressed apical tergal margins dark yellowish to dark reddish translucent (Fig. 1C, D). T1 in anterior half densely covered with short appressed white to yellowish-white hair, T1 entirely and T2 on disc medially with sparse long and erect, and T2 anteriorly sparsely covered with long, erect white to yellowish-white hairs (Fig. 1C, D); apical tergal hair bands dense and broad, white to yellowish-white (Fig. 1C, D). Apical sternal hair fringes medially significantly shorter than laterally (Fig. 1B). Terga densely and finely punctate (i ≤ d), interspace smooth and shiny (Fig. 1C, D). ***Terminalia***. Genitalia and S7 as illustrated (Fig. 1E, F).

#### Etymology.

The species name perplexus refers to the Latin term for “enigmatic” or “ambiguous”, since the specimens could potentially represent the unknown male of *C. penulatus* Noskiewicz, 1936 (see below comment under “Remarks”).

#### General distribution.

Only known from the type locality in southern Azerbaijan.

#### Floral hosts.

Unknown.

#### Seasonal activity.

May.

#### Remarks.

It cannot be ruled out that the specimens described here as a new species is the unknown male of *C. penulatus*. They belong to the same species group and were found in the same region (valley of the river Ares, formerly Araxes). The type specimen of *C. penulatus*, a single female, was placed in Noskiewicz' collection (Noskiewicz 1936: 287) but the specimen is not there (Wanat et al. 2014). The type is probably lost, so its identity cannot be determined with certainty at present.

##### *Colletes fodiens* species group

### Colletes edentulus

Taxon classificationAnimaliaHymenopteraColletidae

Noskiewicz, 1936

8019CADE-E420-5425-919D-B8FA469439EB

Colletes edentulus Noskiewicz, 1936: 329–330, ♂. Holotype: ♂, Araxestal, Azerbaijan.

#### Material examined.

**Azerbaijan. Nakhchivan Autonomous Republic** • 1 ♂, Ordubad, Behrud, 39°04'N, 45°52'E, 1345 m, 7.VIII.2020, MM [FSCV]; • 1 ♂, Shakhbuz, Bichenek, 39°31'N, 45°46'E, 2000 m, 14.VII.2021, MM [FSCV].

#### Distribution.

Russia (North Caucasus), Turkey, Azerbaijan (including *Nakhchivan Autonomous Republic), Turkmenistan.

### Colletes glaber

Taxon classificationAnimaliaHymenopteraColletidae

Warncke, 1978

982CB713-B1BC-53E8-9B2B-195FA405D420

Figs 2, 3, 4

Colletes glaber Warncke, 1978: 350–352, ♂. Holotype: ♂ [Warncke 1978: 352 mentioned a ♀ as the Holotype but in fact it is a ♂], Şereflikoçhisar, Turkey (OÖLM), examined.

#### Material examined.

**Azerbaijan. Nakhchivan Autonomous Republic** • Julfa, Daridagh, 39°03'N, 45°40'E, 1100 m, 26.V.2022, 1 ♀, MM [FSCV].

#### Distribution.

Jordan, Russia (North Caucasus), Turkey, Armenia, *Azerbaijan (Nakhchivan Autonomous Republic).

#### Remarks.

**Figure 2.**
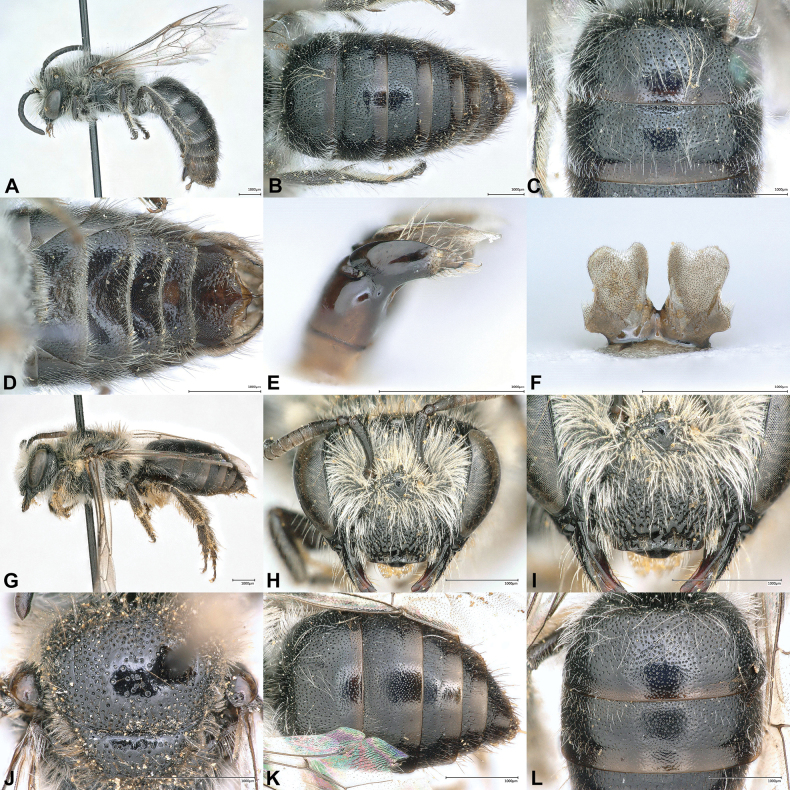
*Colletes glaber* Warncke, 1978, type specimens from Şereflikoçhisar [38.56°N, 33.33°E], Turkey. Holotype male (**A–F**) and paratype female (**G–L**). **A**. Habitus, lateral view; **B**. Metasoma, dorsal view; **C**. T1–T2, dorsal view; **D**. Metasoma, ventral view; **E**. Gonostylus, lateral view; **F**. S7, dorsal view; **G**. Habitus, lateral view; **H**. Head, frontal view; **I**. Clypeus, frontal view; **J**. Scutum, dorsal view; **K**. Metasoma, dorsal view; **L**. T1–T2, dorsal view.

**Figure 3.**
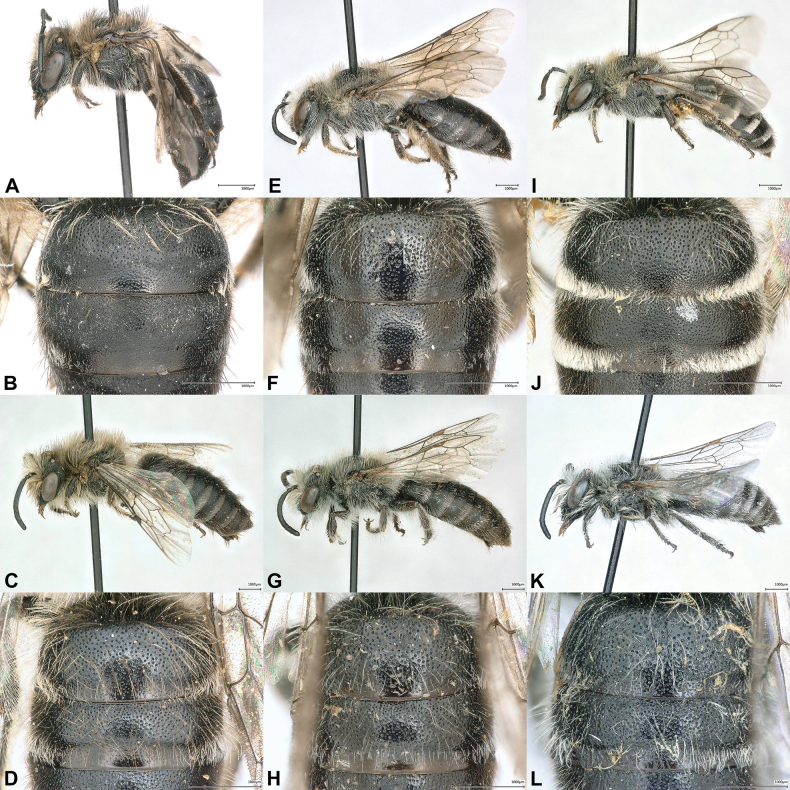
*Colletes glaber* Warncke, 1978, intraspecific (geographical) variation of pilosity and integument. Specimens from Russia (Dagestan, Tekipirkent, 41°20'N, 47°52'E) (**A–D**), from Turkey (Güzelsu: 38°19'N, 43°33'E) (**E–H**) and from Jordan (80 km NE of Aqaba, 29°50'N, 35°20'E) (**I–L**). **A, E, I**. Female habitus, lateral view; **B, F, J**. Female T1–T2, dorsal view; **C, G, K**. Male habitus, lateral view; **D, H, L**. Male T1–T2, dorsal view.

**Figure 4.**
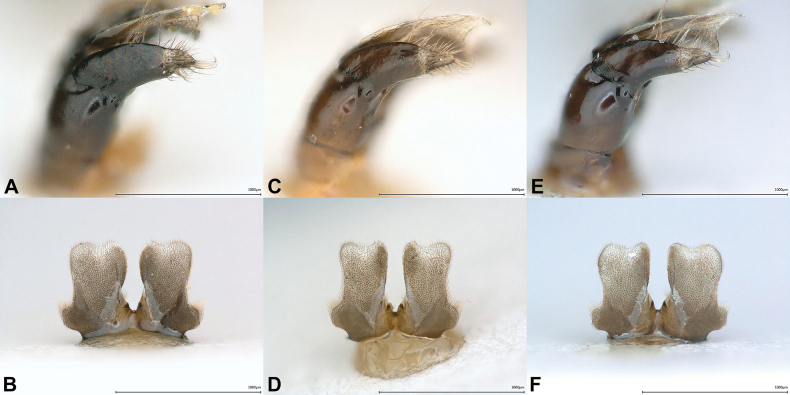
Males of *Colletes glaber* Warncke, 1978, intraspecific (geographical) variation of pilosity and integument. Specimens from Russia (Dagestan, Tekipirkent, 41°20'N, 47°52'E) (**A, B**), from Turkey (Güzelsu: 38°19'N, 43°33'E) (**C, D**) and from Jordan (80 km NE of Aqaba, 29°50'N, 35°20'E) (**E, F**). **A, C, E**. Gonostylus, lateral view; **B, D, F**. S7, dorsal view.

**Figure 5.**
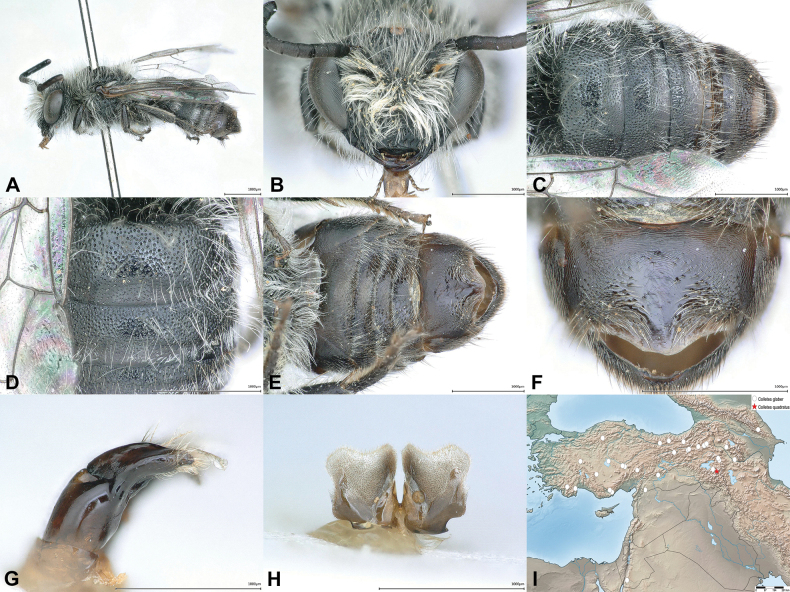
*Colletes quadratus* sp. nov., male, holotype. **A**. Habitus, lateral view; **B**. Head, frontal view; **C**. Metasoma, dorsal view; **D**. T1–T2, dorsal view; **E**. Metasoma, ventral view; **F**. S6, ventral view; **G**. Gonostylus, lateral view; **H**. S7, dorsal view; **I**. Distribution records of *Colletes glaber* Warncke, 1978 (open circle) and *C. quadratus* sp. nov. (red star).

The intraspecific variation of *C. glaber* has been known for some time. The variability of the male S7 and gonostylus is particularly striking, as its shape and hairiness are usually stable and species-specific. It therefore seemed possible that this species, which is predominantly found in the mountains of Turkey, could be a complex of closely related taxa. The first record of *C. glaber* in Azerbaijan was taken as an opportunity to try and clarify the long-standing taxonomic uncertainties around this species and to re-examine the available specimens (in total 65 females and 18 males). Type material was also included in the study and the male Holotype and a female Paratype, both collected at the same locality and date (Turkey: Şereflikoçhisar, 17 May 1970), are photographically documented in detail (Figs 2, 3, 4) as a reference. Data on the records of all examined specimens of *C. glaber*, most of which are unpublished, are listed below and the distribution of the species is summarised in Fig. 5I. Information on the type material is also included, since Warncke (1978) only provided incomplete data. It was found that the holotype of *C. glaber* is a male and not a female, as erroneously stated by Warncke (1978: 352).

#### Type material (6 specimens).

**HOLOTYPE**: ♂. **Turkey**. • Şereflikoçhisar / Ankara [38.56°N, 33.33°E], 17.5.1970, Warncke (OÖLM, Coll. Warncke). **PARATYPES. Turkey**. • Kilis / Gaziantep [36.43°N, 37.07°E], 13.5.1975, 1 ♀, K. Warncke (OÖLM, Coll. Warncke); • Sille / Konya [37.55°N, 32.25°E], 9.-17.6.1975, 1 ♀, J. Heinrich (OÖLM, Coll. Warncke); • same data as for the Holotype, 1 ♀ (OÖLM, Coll. Warncke); • Tanyeri / Erzincan [39.36°N, 39.51°E], 13.6.1973, 1 ♀, Kl. Warncke (OÖLM, Coll. Warncke); • Horasan / Arastal [40.02°N, 42.09°E], 14.6.1973, 1 ♀, Kl. Warncke (OÖLM, Coll. Warncke).

#### Additional material examined (76 specimens).

**Jordan**. • 80 km NE Aqaba (Straße nach Amman) [29.50°N, 35.20°E], 13.IV.1989, 2 ♂, J. Gusenleitner (OÖLM); • same data as for preceding, 15.IV.1989, 3 ♀, (OÖLM). **Turkey**. • 30 km N of Silifke, Uzuncabure [36.34°N, 33.54°E], 28.V.1996, 14 ♀, 2 ♂, M. Halada (OÖLM, RCMK); • 40 km E of Mut, Cornelek [36.42°N, 33.41°E], 29.V.1996, 4 ♀, M. Halada (OÖLM, RCMK); • Antalya, ca. 6 km E of Saklikent [36.50°N, 30.24°E], 1.VI.2009, 1 ♀, 1 ♂, J.S. Ascher, J.G. Rozen, H. Özbek (AMNH); • Burdur, around Dirimli, 36°55'56"N, 29°38'39"E, 1350 m, 6.VI.2006, 1 ♀, E. Scheuchl (RCMK); • Kuyucak, Adiyaman [37.51°N, 38.20°E], 8.VI.1998, 2 ♀, M. Halada (OÖLM); • Sille bei Konya [37.55°N, 32.25°E], 10.VI.1966, 1 ♀, H.H.F. Hamann (OÖLM); • Ihara valley [38.15°N, 36.18°E], 13.VI.2008, 1 ♀, M. Kafka (OÖLM); • Güzelsu env., 40 km south-east of Van [43.33°N, 38.19°E], 2000 m, 7.VI.2001, 10 ♀, 4 ♂, K. Denes sen. (OÖLM, RCMK); • Topuzdagi Gecidi, 38°31.63'N, 35°07.01'E, 1500 m, 18.VI.2000, 1 ♀, M. & O. Niehuis (RCMK); • Ürgüp [38.37°N, 34.54°E], 17-19.VI.1976, 1 ♀, Jos. Heinrich (OÖLM); • Afyon, Koroglu mountain pass, 38°55'20"N, 30°53'40"E, 1295 m, 21.VI.2006, 2 ♀, E. Scheuchl (PCES); • 40 km E of Muradiye [39.00°N, 44.10°E], 2200 m, 5.VII.2000, 2 ♀, M. Halada (RCMK); • 40 km NE of Muradiye [39.10°N, 44.01°E], 2200 m, 5.VII.2000, 3 ♀, M. Halada (OÖLM); • Erzurum, 15 km SW, Tekederesi env. [39.48°N, 41.10°E], 2300 m, 2.VII.2001, 1 ♀, M. Fikacek, J. Hajek & J. Straka (RCMK); • Erzurum Prov., S side of Palandöken Mtn. [39.51°N, 41.17°E], 5.VII.2007, 1 ♀, J.S. Ascher, J.G. Rozen, H. Özbek (AMNH); • Horasan, 18 km E of Delibaba [40.02°N, 42.09°E], 25.VI.1993, 1 ♀, 4 ♂, Jirousek (PCMS, RCMK); • 20 km E of Karakurt, Arastal [40.06°N, 42.46°E], 12.VI.1977, 3 ♀, K. Warncke (OÖLM); • 50 km S of Kara, Pasli [40.17°N, 42.56°E], 1.VII.1997, 1 ♀, M. Halada (OÖLM); • Giresun, 8-15 km NE of Sebin Karahisar [40.19°N, 38.30°E], 1100 m, 6.VI.1988, 1 ♀, H.v. Oorschot & H. Wiering (NBCL); • Erzurum, Pazaryolu [40.25°N, 40.46°E], 27.VI.2008, 1 ♀, J.G. Rozen, H. Özbek (AMNH); • Gümüshane, 28-42 km N of Terssundagi Gecidi [40.29°N, 39.21°E], 1300 m, 20.VI.1988, 2 ♀, H.v. Oorschot & H. Wiering (NBCL, RCMK); • Cankin, between Sacakbeli pass, 40°41'53"N, 33°00'46"E, 1473 m, 18.VI.2006, 1 ♀, 1 ♂, E. Scheuchl (RCMK). **Azerbaijan**. • Data see above. **Armenia**. • Vedi – Chozrov [40.00°N, 44.50°E], 4.VI.2003, 1 ♀, Mucka (RCMK). **Russia**. • Tekipirkent, 41°20'18"N, 47°52'32"E, 29.VI.2023, 1 ♀, 2 ♂, M. Proshchalykin, A. Fateryga (FSCV, RCMK).

Examination of the specimens above revealed that significant variation exists in the coloration and extent of body hair, punctation of metasomal terga, shape of the male S7 and gonostylus. For both sexes, there is a clear trend toward longer and darker body hair from south to north (Fig. 3A, C, E, G, I, K). In the north, even blackish hairs are admixed on the vertex and on the dorsal side of the mesosoma (Fig. 3A, C), which are absent in specimens collected further south (Fig. 3E, G, I, K). Apical tergal hair bands are distinctly more extensive and denser in the south (Fig. 3B, D, F, H, J, L), which is particularly apparent in females from Jordan (Fig. 3J). The variation observed in the punctation of the terga (Figs 2C, 2L, 3B, 3D, 3F, 3H, 3J, 3L) and the shape of S7 (Figs 2F, 4B, 4D, 4F) and the gonostylus (Figs 2E, 4A, 4C, 4E) does not seem to follow any general geographical pattern, but rather seems to be specific for a region. It appears that the morphological variability of *C. glaber*, that also exists within populations, is a continuum in which the different character states transition into one another in a geographical mosaic. Thus, it is currently not possible to morphologically delineate additional taxa based on the available material. However, genetic analyses, which were not feasible within the scope of this study, and further specimens may require a reassessment of the situation in the future.

The only exception is a single male from the high mountains in southeastern Turkey, at the eastern edge of the distribution of *C. glaber*, that is clearly outside the variation range of this species. The specimen was also collected in higher altitude (> 2500 m) than *C. glaber* specimens and is described below as a new species.

### Colletes quadratus

Taxon classificationAnimaliaHymenopteraColletidae

Kuhlmann sp. nov.

A7B00AB8-4ABE-5308-BAD3-F0DCC51D4656

https://zoobank.org/D02474FB-38D5-4B91-AE67-C9196C658890

Fig. 5A–H

#### Type material (1 specimen).

***Holotype***: ♂. **Turkey**. • “Türkei, Hoşop/Başkale, Güzeldere-Paß E [38.10°N, 43.56°E], 2500–2600 m, 9.7.1984, leg. A.W Ebmer” (RCMK).

#### Diagnosis.

The new species is closely related to *C. glaber* in the *C. fodiens* species group that is characterised in the male sex by the following character combination (Noskiewicz 1936): malar area short, hind basitarsus at least slightly broadened, discs of metasomal terga with long erect hairs, apical hair fringes of metasomal sterna thin and medially interrupted, S6 laterally with small tooth or bulge, covered with a hair brush or hair patch that is usually pointing backwards. The males of both species differ in the following characters: Scutum and metasomal terga strongly convex (*C. glaber*: scutum and metasomal terga less convex, more flattish), punctation on discs of metasomal terga fine and dense (i < 0.5d) (Fig. 5C, D) (*C. glaber*: punctation coarser and sparser (i = 0.5–1d); Figs 2B, 2C, 3D, 3H, 3L), marginal zone of terga completely finely and shallowly punctate and / or shagreened, matt (Fig. 5C, D) (*C. glaber*: at least on T2 and following terga a narrow apical margin of the marginal zone impunctate, polished and shiny; Figs 2B, 2C, 3D, 3H, 3L), S7 approx. as long as broad (Fig. 5H) (*C. glaber*: S7 always distinctly longer than broad; Figs 2F, 4B, 4D, 4F), gonostylus slightly shorter than its basal width (Fig. 5G) (*C. glaber*: gonostylus always shorter than its basal width; Figs 2E, 4A, 4C, 4E).

#### Description.

**Female**. Unknown.

**Male**. Body length = 7.0 mm (Habitus Fig. 5A). ***Head***. Head distinctly wider than long (width to length ratio: 1.4: 1) (Fig. 5B). Integument black except tip of mandible partly dark reddish-brown. Face densely covered with long, greyish-white, erect hairs (Fig. 5B). Malar area medially ~ 1/2 as long as width of mandible base, finely striate. Antenna dorsally dark blackish-brown to black, ventrally slightly lighter; F1 as long as wide (length to width ratio: 1: 1). ***Mesosoma***. Integument black. Dorsolateral angle of pronotum rectangular and rounded. Mesoscutal disc moderately densely punctate (i = 1–2d), interspace smooth and shiny. Scutellum with a slightly denser punctation than mesoscutal disc. Mesoscutum, scutellum, metanotum, mesepisternum and propodeum covered with long, greyish-white, erect hair (Fig. 5A). ***Wings***. Transparent, membrane very light brown; wing venation dark brown. ***Legs***. Integument dark brown to black; last tarsal segments dark reddish-brown. Hind basitarsus apically slightly broadener, ~ 4× as long as apically wide. Vestiture greyish-white. ***Metasoma***. Integument black; depressed apical tergal margins partly dark reddish-brown translucent (Fig. 5C, D). T1–T6 sparsely covered with erect long but successively shorter and darker (from greyish-white to brown) hairs; apical tergal hair bands sparse and narrow (Fig. 5C, D). Terga densely and relatively finely punctate (i ≤ 0.5d), interspace smooth and shiny (Fig. 5C, D); depressed marginal zones of terga completely finely and shallowly punctate and / or shagreened, matt (Fig. 5C, D). Structure and pilosity of sterna as in Fig. 5E, F. **Terminalia**. Genitalia and S7 as illustrated (Fig. 5G, H).

#### Etymology.

The species name refers to the almost square shape of S7 (Fig. 5H).

#### General distribution.

Only known from the type locality in the Southeastern Taurus Mountains of Turkey (Fig. 5I).

#### Floral hosts.

Unknown.

#### Seasonal activity.

July.

##### *Colletes senilis* species group

### Colletes senilis

Taxon classificationAnimaliaHymenopteraColletidae

(Eversmann, 1852)

D99A62E7-5D41-5F98-92A1-0CD5896FB04C

Andrena senilis Eversmann, 1852 (nec Smith 1879): 21–22, ♀, ♂. Lectotype: ♂, Casanensi [Tatarstan Republic, Russia], designated by Proshchalykin et al. 2017: 15.

#### Material examined.

**Azerbaijan. Nakhchivan Autonomous Republic** • Kengerli, Tezekend, 39°24'N, 45°17'E, 1130 m, 4.V.2021, 4 ♀, 2 ♂, MM [FSCV/RCMK].

#### Distribution.

Greece, Iran, Italy, Macedonia, Russia (European part, Urals), Turkey, Armenia, Azerbaijan (including *Nakhchivan Autonomous Republic), Kazakhstan.

##### *Colletes cunicularius* species group

### Colletes caskanus

Taxon classificationAnimaliaHymenopteraColletidae

(Strand, 1919)

E04C4FBC-0A80-5FC2-AF5F-BD8FCDBE7541

Andrena caskana Strand, 1919: 45, ♀, ♂. Syntypes: ♀♀, ♂♂, Caska, Macedonia.

#### Material examined.

**Azerbaijan. Nakhchivan Autonomous Republic** • Ordubad, Bilav, 39°03'N, 45°49'E, 1050 m, 28.IV.2021, 1 ♀, MM [FSCV]; • Julfa, Daridagh, 39°03'N, 45°40'E, 1100 m, 20.IV.2022, 2 ♂, MM [FSCV/ZISP]; • Shakhbuz, Batabat, 39°31'N, 45°47'E, 2100 m, 2 ♂, 27.V.2022, MM [FSCV/RCMK].

#### Distribution.

Croatia, Serbia, Bulgaria, Greece, Macedonia, Turkey, Syria, Jordan, *Azerbaijan (Nakhchivan Autonomous Republic).

##### *Colletes marginatus* species group

### Colletes hethiticus

Taxon classificationAnimaliaHymenopteraColletidae

Warncke, 1978

17C17CE3-2611-5EFF-8A42-C2F61C40A30E

Colletes marginatus hethiticus Warncke, 1978: 358–359, ♀, ♂. Holotype: ♂, Konya, Turkey.

#### Material examined.

**Azerbaijan. Nakhchivan Autonomous Republic** • Julfa, Gazanchi, 39°13'N, 45°41'E, 27.VII.2018, 1 ♀, MP, HA, MM [FNCV]; • Julfa, Ashabi-Kahf, 39°13'N, 45°35'E, 1330 m, 28.VI.2019, 3 ♂, MM [FSCV/RCMK]; • Nakhchivan, 39°13'N, 45°24'E, 890 m, 26.VI.2019, 1 ♂, MM [FSCV]; • Ordubad, Ustupu, 39°02'N, 45°54'E, 1495 m, 16.VI.2024, 2 ♀, 1 ♂, MP, MM [FSCV/RCMK]; • Ordubad, Channab, 38°59'N, 45°53'E, 1090 m, 16.VI.2024, 2 ♀, MP, MM [FSCV/RCMK]; • Ordubad, Anagut, 38°58'N, 45°56'E, 1250 m, 9.VI.2021, 1 ♂, MM [RCMK]; • Babek, Shikhmakhmud, 39°15'N, 45°25'E, 940 m, 24.VI.2021, 1 ♀, 2 ♂, MM [FSCV/RCMK].

#### Distribution.

Romania, Bulgaria, Russia (North Caucasus), Turkey, Azerbaijan (including *Nakhchivan Autonomous Republic).

##### *Colletes cariniger* species group

### Colletes cariniger

Taxon classificationAnimaliaHymenopteraColletidae

Pérez, 1903

A09E2C3F-2407-51F5-9A3B-502A74B0152F

Colletes cariniger Pérez, 1903: 228, ♂. Syntypes: ♂♂, Malatia, Syria.

#### Material examined.

**Azerbaijan. Nakhchivan Autonomous Republic** • Kengerli, Garabaglar, Asni, 39°26'N, 45°12'E, 1390 m, 19.IV.2018, 2 ♂, MM [FNCV]; • Kengerli, Tezekend, 39°24'N, 45°17'E, 1130 m, 4.V.2021, 4 ♀, MM [FSCV/ZISP]; • Julfa, Daridagh, 39°03'N, 45°40'E, 1100 m, 26.V.2022, 1 ♀, MM [FSCV].

#### Distribution.

Egypt, Bulgaria, Greece, Israel, Jordan, Lebanon, Libya, Syria, Turkey, Azerbaijan (including *Nakhchivan Autonomous Republic).

##### *Colletes albomaculatus* species group

### Colletes albomaculatus

Taxon classificationAnimaliaHymenopteraColletidae

(Lucas, 1849)

E035AAD0-6E4A-5250-9D61-CE1FABE09D94

Halictus albomaculatus Lucas, 1849: 183, ♀. Syntypes: ♀♀, Algeria.

#### Material examined.

**Azerbaijan. Nakhchivan Autonomous Republic** • Shakhbuz, Kulus, 39°21'N, 45°37'E, 1395 m 25.VI.2022, 1 ♀, 1 ♂, MM [FSCV]; • ibid., 18.VI.2024, 1 ♀, MP, MM [FSCV]; • Babek, Payiz, 39°26'N, 45°22'E, 1230 m, 28.VI.2024, 1 ♀, MP, MM [FSCV]; • Ordubad, Channab, 38°59'N, 45°53'E, 1090 m, 16.VI.2024, 1 ♀, MP, MM [FSCV]; • Ordubad, Ustupu, 39°02'N, 45°54'E, 1495 m, 16.VI.2024, 1 ♀, MP, MM [FSCV]; • Shakhbuz, Badamli, 39°28'N, 45°33'E, 1290 m, 17.VI.2024, 2 ♀, MP, MM [FSCV]; • Shakhbuz, Kolani, 39°26'N, 45°39'E, 1330 m, 21.V.2025, 1 ♀, 12 ♂, MP, MM [FSCV/ZISP]; • Shakhbuz, Zarnatun, 39°28'N, 45°43'E, 1550 m, 21.V.2025, 3 ♂, MP, MM [FSCV].

#### Distribution.

North Africa, Europe, Russia (North Caucasus, European part), Turkey, Syria, Jordan, Armenia, Georgia, Azerbaijan (including *Nakhchivan Autonomous Republic), Iran, Tajikistan, Kyrgyzstan.

##### *Colletes nasutus* species group

### Colletes nasutus

Taxon classificationAnimaliaHymenopteraColletidae

Smith, 1853

65057677-9AC0-5082-A25A-5B7DF35D728B

Colletes nasutus Smith, 1853: 3, ♀, ♂. Syntypes: ♀♀, ♂♂, Poland.

#### Material examined.

**Azerbaijan. Nakhchivan Autonomous Republic** • Shakhbuz, Bichenek, 39°31'N, 45°46'E, 2000 m, 3.VII.2019, 2 ♀, 3 ♂, MM [FSCV]; • Shakhbuz, Kechili, 39°22'N, 45°43'E, 1800 m, 26.VI.2020, 1 ♂, MM [FSCV]; • Kengerli, Chalkhangala, 39°26'N, 45°15'E, 1440 m, 19.VI.2024, 2 ♀, MP, MM [FSCV]; • Shakhbuz, Zarnatun, 39°28'N, 45°43'E, 1550 m, 21.VI.2024, 1 ♀, MP, MM [FSCV]; • Ordubad, Unus, 39°01'N, 45°59'E, 1680 m, 22.VI.2024, 2 ♀, 4 ♂, MP, MM [FSCV/RCMK]; • Julfa, Lakadagh, 39°17'N, 45°50'E, 1760 m, 24.VI.2024, 1 ♀, MP, MM, [FSCV]; • Sharuz, Shahbulag, 39°38'N, 45°08'E, 1210 m, 26.V.2025, 2 ♀, 8 ♂, MP, MM [FSCV/RCMK].

#### Distribution.

Europe, Syria, Turkey, Iran, Armenia, Azerbaijan (including *Nakhchivan Autonomous Republic).

## Discussion

In the present study, we list new records for 13 species of the genus *Colletes* from various localities in the Nakhchivan Autonomous Republic and describe a fourteenth. Together with published records, 25 *Colletes* species are currently known to occur in the Nakhchivan Autonomous Republic and 43 species throughout Azerbaijan. For comparison, 45 species have been recorded from Iran, 28 species from Armenia, 17 species from Georgia, and 25 species from Dagestan (Russia) (Kuhlmann and Proshchalykin 2016; Kuhlmann et al. 2023; Proshchalykin and Kuhlmann 2025). The Nakhchivan Autonomous Republic’s *Colletes* fauna is quite original; of the 43 known *Colletes* species in Azerbaijan, seven species are found only in this territory, including three endemic species (*C. dlusskyi* Kuhlmann & Proshchalykin, 2015, *C. jovel* Kuhlmann & Proshchalykin, 2016 and *C. perplexus* sp. nov.).

The intraspecific variability of *C. glaber* is here documented for the first time. Identifiable was a general, presumably climate-driven, trend of hair getting longer and darker from south to north. No (geographical) patterns were apparent in the remaining variation. These appear to be local or regional variations that are not uncommon in species distributed across mountain regions. The study led to the discovery of the previously unrecognised new species *C. quadratus* sp. nov. found in the hardly accessible, understudied higher altitudes of the mountains of southeastern Turkey.

## Supplementary Material

XML Treatment for Colletes

XML Treatment for Colletes hylaeiformis

XML Treatment for Colletes eous

XML Treatment for Colletes idoneus

XML Treatment for Colletes maidli

XML Treatment for Colletes wollmanni

XML Treatment for Colletes perplexus

XML Treatment for Colletes edentulus

XML Treatment for Colletes glaber

XML Treatment for Colletes quadratus

XML Treatment for Colletes senilis

XML Treatment for Colletes caskanus

XML Treatment for Colletes hethiticus

XML Treatment for Colletes cariniger

XML Treatment for Colletes albomaculatus

XML Treatment for Colletes nasutus

## Additional information

### Use of AI

No use of AI was reported.

### Author ORCIDs

Michael Kuhlmann https://orcid.org/0000-0003-3664-6922

Maxim Yu. Proshchalykin https://orcid.org/0000-0001-7870-8226

Mahir M. Maharramov https://orcid.org/0000-0002-4130-7071

### Data availability

All of the data that support the findings of this study are available in the main text.
